# Population-Level Benefits from Providing Effective HIV Prevention Means to Pregnant Women in High Prevalence Settings

**DOI:** 10.1371/journal.pone.0073770

**Published:** 2013-09-16

**Authors:** Dobromir Dimitrov, Marie-Claude Boily, Jeannie Marrazzo, Richard Beigi, Elizabeth R. Brown

**Affiliations:** 1 Vaccine & Infectious Disease Division, Fred Hutchinson Cancer Research Center, Seattle, Washington, United States of America; 2 Department of Infectious Disease Epidemiology, Faculty of Medicine, Imperial College London, London, United Kingdom; 3 Department of Medicine, University of Washington, Seattle, Washington, United States of America; 4 Magee-Womens Hospital, University of Pittsburgh Medical Center, Pittsburgh, Pennsylvania, United States of America; 5 Department of Applied Mathematics, University of Washington, Seattle, Washington, United States of America; 6 Department of Biostatistics, University of Washington, Seattle, Washington, United States of America; UCL Institute of Child Health, University College London, United Kingdom

## Abstract

**Background:**

HIV prevalence among pregnant women in Southern Africa is extremely high. Epidemiological studies suggest that pregnancy increases the risk of HIV sexual acquisition and that HIV infections acquired during pregnancy carry higher risk of mother-to-child transmission (MTCT). We analyze the potential benefits from extending the availability of effective microbicide to pregnant women (in addition to non-pregnant women) in a wide-scale intervention.

**Methods and Findings:**

A transmission dynamic model was designed to assess the impact of microbicide use in high HIV prevalence settings and to estimate proportions of new HIV infections, infections acquired during pregnancy, and MTCT prevented over 10 years. Our analysis suggests that consistent use of microbicide with 70% efficacy by 60% of non-pregnant women may prevent approximately 40% and 15% of new infections in women and men respectively over 10 years, assuming no additional increase in HIV risk to either partner during pregnancy (RR_HIV/preg_ = 1). It may also prevent 8–15% MTCT depending on the increase in MTCT risk when HIV is acquired during pregnancy compared to before pregnancy (RR_MTCT/preg_). Extending the microbicides use during pregnancy may improve the effectiveness of the intervention by 10% (RR_HIV/preg_ = 1) to 25% (RR_HIV/preg_ = 2) and reduce the number of HIV infections acquired during pregnancy by 40% to 70% in different scenarios. It may add between 6% (RR_HIV/preg_ = 1, RR_MTCT/preg_ = 1) and 25% (RR_HIV/preg_ = 2, RR_MTCT/preg_ = 4) to the reduction in the residual MTCT.

**Conclusion:**

Providing safe and effective microbicide to pregnant women in the context of wide-scale interventions would be desirable as it would increase the effectiveness of the intervention and significantly reduce the number of HIV infections acquired during pregnancy. The projected benefits from covering pregnant women by the HIV prevention programs is more substantial in communities in which the sexual risk during pregnancy is elevated.

## Introduction

Four clinical trials have shown that oral and topical pre-exposure prophylaxis (PrEP) based on tenofovir may be effective in preventing HIV transmission [1]–[4]. Although these positive findings were not confirmed by other trials [5], [6] a tenofovir based product (Truvada) has been approved for PrEP use in the US [7] and recommended as a preventive option to MSM in South Africa [8]. It is unclear when PrEP will be accessible to heterosexual men and women but it is clear that considerable safety data will be needed before it is explicitly recommended for use by pregnant women.

Pregnancy has been a consistent exclusion criterion for participation in oral and topical PrEP efficacy trials [1], [3], [9], [10], and participants who become pregnant during follow up are temporarily removed from product use due to safety concerns. To address this lack of safety data, numerous studies have been undertaken to gather data that will inform safe use of HIV prevention agents in pregnancy. The first clinical study of single-dose Tenofovir gel applied during late pregnancy reported no adverse effects despite evidence of a trace of absorption of the product in the maternal and cord blood [11]. A subsequent trial is currently enrolling HIV negative women in the third trimester of pregnancy to test the expanded safety of multiple dosing of Tenofovir gel [12]. A separate observational cohort investigation is concurrently enrolling women who have become pregnant during microbicide or oral PrEP trials to investigate inadvertent exposure to HIV prevention study agents early in gestation [13]. If these parallel studies in pregnant women demonstrate safety they might allow women who become pregnant to receive faster access to the product after its approval.

Published analyses suggest that pregnant women are among those who can highly benefit from novel HIV prevention methods. Recent studies report that the risk of HIV acquisition during pregnancy is elevated for pregnant women and even for their male partners in serodiscordant couples [14], [15]. Gray and colleagues estimated that the HIV incidence rate during pregnancy is 2.3% compared to 1.1% in the non-pregnant and non-lactating women in Rakai, Uganda [14]. They suggested that increased risk during pregnancy might be attributable to hormonal changes rather than sexual risk behavior. A study conducted by the Partners in Prevention HSV/HIV Transmission Study Team among serodiscordant couples from seven African countries reported that the HIV incidence in women is 7.35% versus 3.01% during pregnant and non-pregnant periods [15]. The HIV incidence of male partners of pregnant and non-pregnant women was estimated at 3.46% and 1.58%, respectively. These investigators concluded that the elevated HIV risk associated with pregnancy may be explained by a combination of behavioral and biological factors. Both studies outlined the need for additional HIV prevention efforts during pregnancy to protect mothers, newborns, and potentially male partners.

High maternal plasma HIV-1 load is a strong predictor for mother-to-child transmission (MTCT) [16]–[18]. Assessing the factors that influence the risk of MTCT in New York, Birkhead and colleagues estimated that maternal HIV acquisition during pregnancy is associated with 15-fold increase in transmission risk [18]. They concluded that avoiding infections during pregnancy must be among the public-health priorities when MTCT is addressed. A separate study investigating the prevalence of acute HIV infections among pregnant women in the US suggests that a substantial proportion of the perinatal transmission in resource-rich settings may be caused by HIV acquisitions during pregnancy [19]. However, a modeling study using data from Zimbabwe projects that less than 6.2% of the infections in infants occur in recently seroconverted mothers [20].

In this study mathematical models are used to estimate the expected population-level benefits from providing effective HIV prevention means to pregnant women in addition to non-pregnant women in high prevalence settings. It is assumed that topical PrEP, with its low level of systemic absorption, will be more appropriate than oral PrEP as a prevention method for healthy pregnant women and therefore we use a wide-scale intervention with vaginal microbicides (VMB) as an example. We assess the cumulative HIV infections prevented in women and men over 10 years, HIV infections during pregnancy prevented, and the fractions of MTCT prevented due to VMB use by pregnant women under various implementation scenarios.

## Methods

### Transmission Model

**Figure 1:**
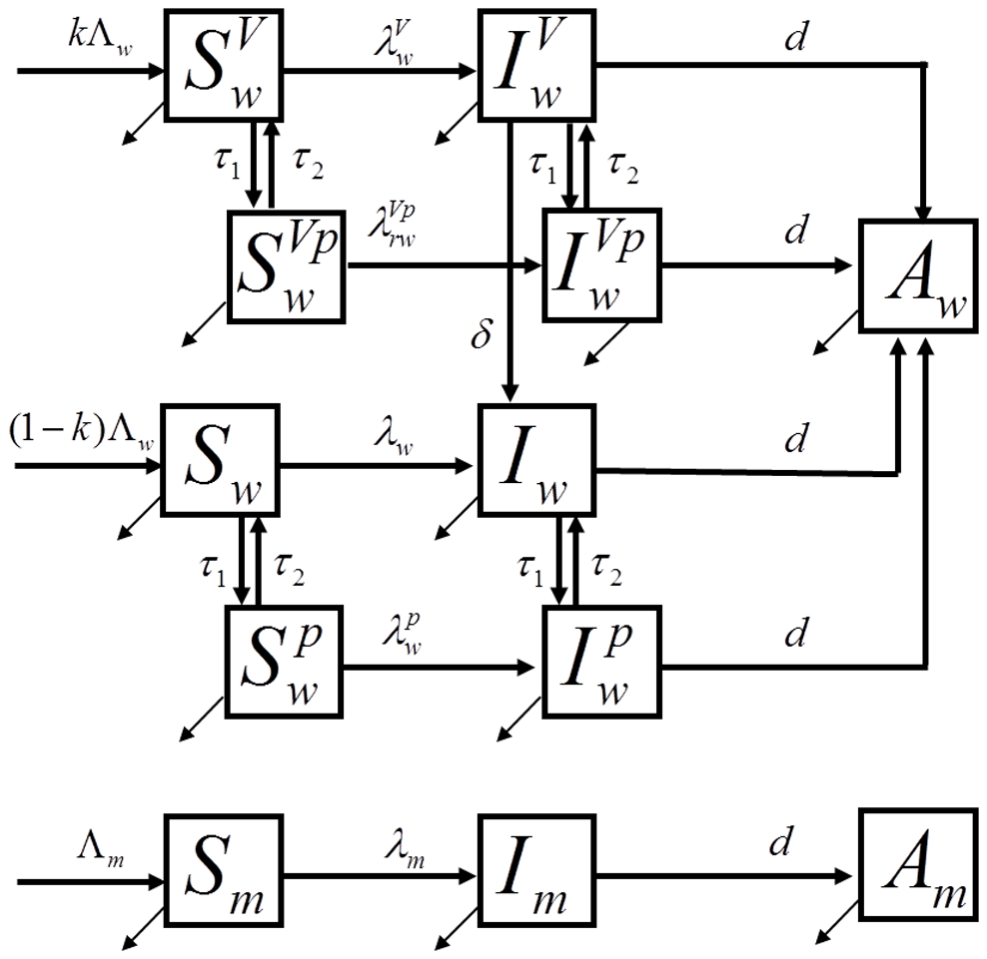
Flow diagram of the model. Simulated population is stratified in compartments by gender as men (subscript m) or women (subscript w) and by HIV status as susceptibles (S), infected with HIV (I) and sexually inactive due to progression to AIDS (A). Female population is additionally stratified by pregnancy status (superscript p indicates pregnancy) and by usage of VMB (superscript p indicates the use of VMB). VMB users strictly follow the prescribed regimen. A complete description of the model including the flow rates and the expressions for the forces of infections (λ) is presented in File S1.

We developed a compartmental mathematical model to study HIV transmission in sexually-active (age 15–49) heterosexual population (Figure 1). The model is designed to determine what would be the difference in HIV transmission if intervention with an effective VMB is rolled out to both pregnant and non-pregnant women compared to non-pregnant women only. The simulated population is divided in compartments by gender as men (subscript g = m) or women (subscript g = w), by HIV status as susceptibles (S), infected (I), and infected individuals who developed AIDS (A). Women are additionally stratified by pregnancy status (superscript p when pregnant) and by VMB use (superscript V for users). Men and women who become sexually active join the community at constant rates that are selected to balance the departure rate in a non-infected population. Adolescents who reach sexual maturity are assumed to become susceptible to HIV. The rates at which men and women acquire HIV-infection are derived from standard binomial models based on the number of sex partners per susceptible person, the number of sex acts per partnership, the fraction of sex acts protected by condoms, the protection provided by VMB (if used) and the HIV acquisition risk per vaginal act for men and women. Women who decide to use VMB are assumed to undergo initial and subsequently annual HIV testing. They are assumed to adhere to VMB use as prescribed, but are removed from VMB if testing positive for HIV. Complete description of the model is given in File S1.

### Epidemic Settings and Intervention Scenarios

We identified 1000 parameter sets which represent current epidemic conditions in South Africa. Behavioral and epidemic parameters are initially varied in experimental parameter ranges representative for the epidemics in Sub-Saharan Africa (see Table 1, part A) and posterior parameter sets are preselected to satisfy the following target criteria: 1) initial HIV prevalence of 15% and 20% among 15–49 year old men and women, respectively [21]; ii) Annual HIV incidence rate between 0.6% and 2.5% [22]; iii) Female incidence rate at least 30% higher than male incidence rate and iv) The absolute difference in HIV prevalence over 5 years remains below 1% (mature epidemics). For each set of epidemic parameters we simulate an HIV epidemic: i) assuming no VMB use; ii) assuming that VMB is used by proportion of the female population during non-pregnant periods only (“restricted” scenario), iii) assuming that VMB is used by the same proportion of the female population without interruption during pregnancy (“choice” scenario) and iv) assuming that VMB is used by a fraction of the female population during non-pregnant periods but by all pregnant women (“targeted” scenario). The effectiveness of the VMB interventions is measured by the cumulative fraction of new HIV infections prevented (women, men and total), HIV infections during pregnancy prevented, and the proportion of MTCT prevented due to VMB use by pregnant women. All intervention outcomes are evaluated over 10 years of VMB use.

**Table 1:** Description and values of the behavioral and epidemic parameters used in the analysis.

Parameter	Description	Parameter range	Ref.
**A. Behavioral and epidemic parameters (pre-intervention)**		**Prior range** 1	
β_w_	Female HIV acquisition risk per vaginal act	0.0019–0.0046	[34]
β_m_	Male HIV acquisition risk per vaginal act	(50–100%) of β_w_	assumed [34]
1/μ	Average time (in years) to remain sexually active	35	[35], [36]
d, d_r_	Annual rates of progression to AIDS for individuals infected with HIV	0.08–0.14	[37], [38]
n_w_,n_m_	Average number of sexual acts per year for women and men	60–100	[39], [40]
ρ_w_, ρ_m_	Average number of sexual partners per year for women and men	1–2	[39], [40]
c	Rate of condom use in general population	20%–60%	[39], [40]
α_c_	Condom efficacy per sex act	0.9	[41]
τ_1_	Annual pregnancy rate	8%–12%	[25]
τ_2_	Annual rate of return from pregnancy (for women)	1.33	calc.
γ	Relative rate of condom use during pregnancy compared to when non-pregnant	0%, 50%, 93%	calc. Fig.2a
r_p_	Relative HIV acquisition risk per sex act during pregnancy compared to when non-pregnant	1.16, 1.50, 2	calc. Fig.2a
**B. Calibrated epidemic data from South Africa**			
*Pw*	Initial HIV-prevalence (women)	20%	[21]
*Pm*	Initial HIV-prevalence (men)	15%	[21]
*Inc*	Fitted HIV-incidence (total)	0.6–2.5%	[22]
*Pr5*	Fitted HIV-prevalence in 5 years (total)	16.5%–18.5%	assumed

1 Ranges for epidemic parameters are sampled uniformly to obtain parameter sets which are filtered to select 1000 epidemics meeting the target criteria about HIV incidence and HIV prevalence.

### VMB Efficacy and Intervention Characteristics

The results from CAPRISA-004 suggest that when used consistently VMB may reduce susceptibility by 54% (95% CI: 4–80%) [1]. In this analysis we explore 50% and 70% reduction in the HIV acquisition per sex act due to VMB use. Although, ARV-based VMB may also reduce the infectiousness of the HIV-positive users we do not consider a reduced risk for the partners of infected VMB users. To ensure comparability, in all scenarios we assume that 60% of the sexually active women start using VMB from the moment of its introduction and follow strictly the prescribed regimen (perfect adherence). A fraction of adolescent women who enter the sexually active population also become VMB users. Although important for the absolute effectiveness of VMB interventions [23] the assumption of perfect adherence is unlikely to affect the relative impact of extending VMB use to pregnant women provided that pregnant and non-pregnant women use VMB with similar consistency. Although pregnancy may be a time when women are more aware of their health and may take measures to increase their personal protection, we assume that this does not result in increased adherence to VMB. Conversely, the recent CAPRISA study looking at pregnancy and study product use suggests that trial participants who did not use product were more likely to become pregnant [24]. The model reflects efforts to limit VMB use by HIV positive women both at the introduction of the intervention and through periodic HIV screening. No changes in sexual behavior (frequency of sex acts, condom use rate) due to the availability of VMB are considered but behavior alterations during pregnant periods are investigated as described in the next section. Description of all intervention parameters and their ranges is presented in Table 2. The effects of key intervention parameters on the modeling results are studied in a multivariate sensitivity analysis (see File S1, Fig. S4).

**Table 2:** Description and values of the intervention parameters used in the analysis.

Parameter	Description	Baseline value (range)^1^	Ref.
k	VMB coverage among adolescent girls reaching sexual maturity	60% (40–80%)	assumed
k_1_	Initial VMB coverage among HIV-negative women	60% (40–80%)	assumed
α_s_	VMB efficacy in reducing susceptibility per act	50%, 70% (40–80%)	assumed [1]
α_i_	VMB efficacy in reducing infectiveness per act	0 (0–50%)	assumed
θ	Prescription rejection rate which measures the reduction in the initial fraction ofHIV-positive compared to HIV-negative women who start using VMB	80% (60–100%)	assumed
δ	Annual withdrawal rate from VMB by HIV+ women as a result of periodic HIV screening	2 (1–3)	assumed

1 Theranges for intervention parameters are used in multivariate sensitivity analysis (see File S1).

### Pregnancy and Risk of HIV Acquisition

**Figure 2:**
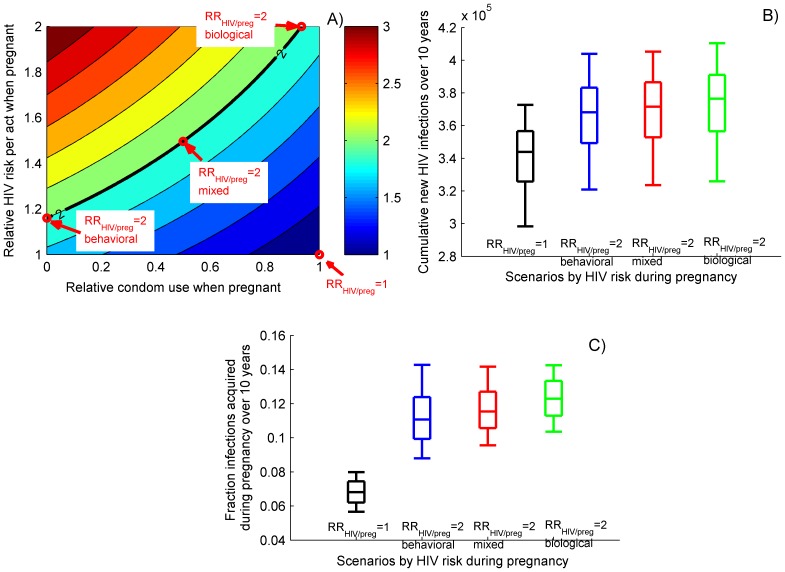
HIV epidemics and risk of HIV acquisition in absence of VMB. A) Relative HIV acquisition risk during pregnancy compared to non-pregnant period of the same length for different combinations of biological susceptibility (relative HIV risk per act compared to when not pregnant) and behavior changes (relative condom use compared to when not pregnant) during pregnancy assuming the same level of sexual activity as before pregnancy. The black line represents combinations which correspond to 2-fold increase in the cumulative HIV risk during pregnancy. Red dots represent the parameter combinations used in the explored scenarios; B) Cumulative number of new HIV infections over 10 years and C) Fraction of the cumulative number of new female HIV infections over 10 years in which HIV is acquired during pregnancy. Presented are scenarios assuming no change in HIV risk during pregnancy (RR_HIV/preg_ = 1, black boxes) and 3 distinct scenarios assuming 2-fold increase in HIV risk during pregnancy (RR_HIV/preg_ = 2, colored boxes). The box plots (median, 5th, 25th, 75th, 95th percentiles) reflect the variation in estimates generated by 1,000 simulations in population with 1 000 000 men and same number of women.

We assume a 8–12% annual pregnancy rate among the sexually active women (15–49 years old) in South Africa which corresponds to 3–4 pregnancies in a lifetime [25]. Two major scenarios with respect to the HIV risk during pregnancy (RR_HIV/preg_) are considered. In the baseline scenario we assume that the probability to be infected with HIV does not change during prenatal periods (RR_HIV/preg_ = 1). In the alternative scenario the HIV risk for pregnant women is doubled (RR_HIV/preg_ = 2) due to increased biological susceptibility and/or behavior alterations (reduced condom use rate) during pregnancy. The cumulative risk per unit time of pregnant and non-pregnant periods is based on standard binomial models which estimate the probability to avoid repeated exposures to HIV (details included in File S1). We identify combinations of relative susceptibility per act and relative condom use when pregnant compared to non-pregnant which correspond to a doubled cumulative risk during pregnancy assuming the same level of sexual activity as before pregnancy (see Fig. 2A). Acknowledging the uncertainty about the mechanism of increased HIV acquisition risk during pregnancy, we explore 3 distinct combinations of biological and behavioral components, assuming that the higher HIV risk during pregnancy is due to i) substantial increase in biological susceptibility and small reduction in condom use (“biological” scenario); ii) moderate (42%) increase in biological susceptibility and 50% reduction in condom use (“mixed” scenario) and iii) small increase (∼16%) in susceptibility and complete elimination of condom use (“behavioral” scenario). VMB interventions under those scenarios are independently evaluated and compared. We also investigate the possibility that the risk for MTCT transmission is elevated when HIV is acquired during pregnancy exploring scenarios in which the relative risk during pregnancy (RR_MTCT/preg_) ranges from 1 to 10. HIV infections of newborn infants are not explicitly modeled but the relative reduction in MTCT transmission is estimated on the basis of reduced number of mothers who are HIV-positive as a result of using VMB.

## Results

In this section we present results from modeling simulations using VMB with 70% efficacy in reducing HIV acquisition risk per vaginal sex act. Similar results on the usage of 50% efficacious VMB are included in File S1 (see Fig. S2 and Fig. S3).

### Elevated HIV Risk during Pregnancy

First, we study the cumulative risk of HIV acquisition during pregnancy for different combinations of elevated biological susceptibility and behavior changes (reduction in condom use) after conception (Fig. 2A) which both may increase the HIV acquisition risk during pregnancy. In the case of complete elimination of condom use and doubled susceptibility per act, the cumulative risk for pregnant women is more than 3 times higher compared to non-pregnant periods. The 2-fold increase reported in some published studies can be obtained by various combinations of biological and behavioral factors (see the black line in Fig. 2A). We simulate the elevated HIV risk during prenatal periods using 3 distinct points along that 2-fold increase curve.

### Epidemic Projections with and without VMB

**Figure 3:**
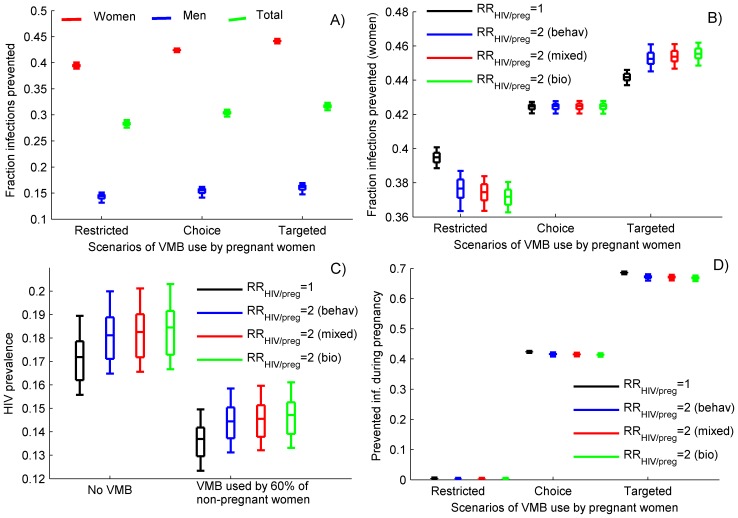
Comparison of the impact of interventions with 70% efficacious VMB used consistently by 60% of the non-pregnant women under different scenarios of VMB use by pregnant women: A) Cumulative fraction of infections prevented over 10 years in women (red), men (blue) and total (green) assuming no change in HIV risk during pregnancy (RR_HIV/preg_ = 1); B) Cumulative fraction of infections prevented in women; C) Projected HIV prevalence after 10 years assuming no VMB use and VMB use by non-pregnant women only. D) Cumulative fraction of infections during pregnancy prevented over 10 years. The scenarios with elevated risk during pregnancy use parameter combinations described in Fig. 2A. The box plots (median, 5th, 25th, 75th, 95th percentiles) reflect the variation in estimates generated by 1,000 different epidemic sets.

If VMB is not available, a total of 345,000 (90% CI 299,000–373,000) new HIV infections are expected over 10 years in a population consisting of 1,000,000 men and same number of women (Fig. 2B) assuming no change in HIV acquisition risk during pregnancy (RR_HIV/preg_ = 1). We estimate that under that scenario approximately 6.8% (90% CI 5.7%–8%) of the newly infected women acquire HIV when pregnant (Fig. 2C) reflecting the maximum proportion of infections that an intervention extended to pregnant women can prevent. Elevated HIV risk during pregnancy (RR_HIV/preg_ = 2) adds up to 10% to the cumulative number of infections and increases the share of the prenatal female infections to 11%–12% with little influence of the mechanism (combination of condom replacement and increased susceptibility) causing the increase in HIV acquisition risk. The overall HIV prevalence in the population after 10 years is projected to be about 17% under scenarios with the same HIV risk for pregnant and non-pregnant women and 18% under scenarios with elevated risk during pregnancy (Fig. 3C).

**Figure 4:**
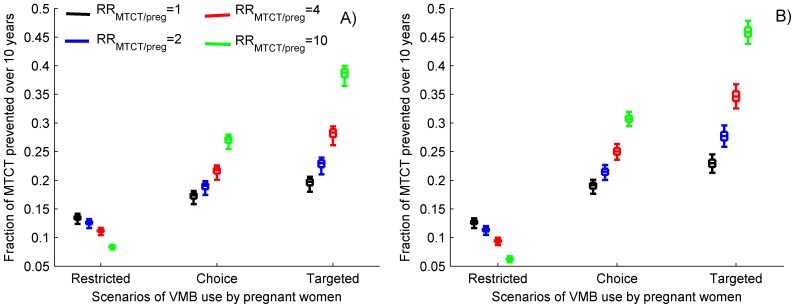
Cumulative fraction of MTCT prevented (right) over 10 years by intervention with 70% efficacious VMB used consistently by 60% of the non-pregnant women under different scenarios of VMB usage by pregnant women assuming: A) no change in HIV risk during pregnancy (RR_HIV/preg_ = 1) and B) HIV risk during pregnancy is elevated (RR_HIV/preg_ = 2, mixed scenario). The colors represent different level of the relative risk of MTCT (RR_MTCT/preg_ ) when HIV is acquired during pregnancy compared to when acquired before pregnancy. The box plots (median, 5th, 25th, 75th, 95th percentiles) reflect the variation in estimates generated by 1,000 different parameters sets.

A consistent use of 70% efficacious VMB by 60% of the non-pregnant women may prevent about 40% of the new HIV infections in women over 10 years, assuming no increase in sexual HIV risk to either partner during pregnancy (RR_HIV/preg_ = 1, Fig. 3A). The projected fraction of infection prevented drops by 2–3 percentage points if the HIV risk during pregnancy is elevated (Fig. 3B). VMB may also contribute to 15% reduction in new male infections over the same period and lead to a decrease by 3 to 4 percentage points in the overall HIV prevalence expected after 10 years of VMB use (Fig. 3C). However, almost no infections acquired during pregnancy will be averted in the scenario excluding pregnant women from VMB use (Fig. 3D) and only between 8% and 15% of the residual MTCT will be averted depending on the relative risk of MTCT in case that HIV is acquired during pregnancy compared to before pregnancy (Fig. 4A).

### Providing VMB to Pregnant Women

The usage of VMB by all pregnant women, may prevent 10–12% more female infections assuming that the HIV risk during pregnancy is not elevated (Fig. 3B, black boxes) and almost 25% if the HIV risk during prenatal periods is doubled (Fig. 3B, colored boxes). Targeted VMB use by pregnant women may have even higher contribution to the overall success of the intervention if the obtained VMB coverage is substantially lower than 60% which we assumed here (see File S1, Fig. S1). More importantly, the access of pregnant women to an effective VMB may reduce the number of HIV infections acquired during pregnancy by 40% to 70% in the “choice” and “targeted” scenarios, respectively (Fig. 3D). As a result, additional 6% (RR_HIV/preg_ = 1) to 10% (RR_HIV/preg_ = 2) absolute reduction in MTCT is projected due to VMB use by pregnant women assuming no increase in the risk of MTCT when HIV is acquired after conception (Fig. 4, black boxes). However, the fraction of MTCT prevented by providing VMB during pregnancy jumps to 40%–45% if the risk for vertical transmission increases by 10-fold, when pregnant women become infected (Fig. 4, green boxes).

## Discussion

Pregnant women have been recognized as a priority group with respect to HIV treatment but their needs for HIV prevention have received less attention. The observed HIV prevalence among pregnant women in many countries in Southern Africa is consistently higher than among general female population with estimates up to 30% for South Africa [26], [27]. Almost 40% of the women who visit prenatal clinics in some provinces of South Africa test HIV positive [27]. HIV infections acquired during pregnancy are likely to remain undiscovered and untreated before delivery. This may seriously increase the risk for both horizontal transmission to sex partners and vertical transmission to newborn infants. Evidence has been presented in recent publications that the HIV acquisition risk during pregnancy is elevated, emphasizing the need to incorporate these data into planning of wide-scale prevention interventions (Fig. 2). Studies which disregard this factor may be underestimating the projected number of infections over 10 years by 10% and the expected HIV prevalence by 1–2 percentage points.

Providing HIV prevention options to women during pregnancy is of critical importance. Unfortunately, pregnant women have been consistently excluded from efficacy trials of oral and topical PrEP agents which only delays the collection of important safety data. The predominant reason for this exclusion centers on concerns for the safety of the fetus [28]. The ethical dilemmas regarding the enrolling pregnant women in biomedical research have long been under discussion with a growing number of researchers advocating for inclusion of pregnant women in clinical trials [29]–[31]. A main justification for inclusion of pregnant women is that preventing the collection of safety and efficacy information during the prenatal periods in controlled clinical settings increases the absolute risk for many more users when the products are licensed, marketed, and then used by the general population. Thus, considerable inadvertent exposure occurs because of use of pharmaceutical products by women unaware of their pregnant status in a less controlled environment than a clinical trial. Recognizing the harm of delaying effective HIV preventive tools for pregnant women and the societal benefits of lessening incident HIV infection during pregnancy, the Microbicide Trial Network has developed a portfolio of clinical trials which will provide data regarding the safety of promising microbicides agents [11]–[13].

Microbicides that protect against HIV and other sexually transmitted disease and show little systemic absorption in the maternal bloodstream may be the optimal prevention option for millions of pregnant and breastfeeding women. We have projected the impact of wide-scale VMB intervention in high-prevalence HIV settings representative for the HIV epidemic in South Africa under different scenarios regarding the access of pregnant women to the VMB. These analyses suggest that the consistent use of effective VMB by large proportion of the non-pregnant women may prevent a substantial fraction of the new infections over 10 years, may reduce the overall HIV prevalence, but will have a little impact on the HIV infections acquired during pregnancy and on the residual MTCT. Including pregnant women into the interventions may have multiple additional beneficial effects. It would increase the effectiveness of the intervention, most significantly when sexual risk during pregnancy is elevated (Fig. 3). More importantly, it may substantially reduce the number of HIV infections acquired during pregnancy and help prevent large proportion of MTCTs which are escaping the current PMTCT efforts (Fig. 4).

The results of this analysis are limited to the specific settings included in the model. The assumptions of instantaneous VMB uptake by a fraction of the female population and perfect compliance with the prescribed VMB regimens may overestimate the overall impact of a VMB intervention but are unlikely to affect the relative contribution of extending the VMB use to pregnant women since the adherence is assumed the same for all women regardless their pregnancy status. Conversely, disregarding a possible reduction of the infectiousness of HIV-positive users of VMB may underestimate the overall impact of VMB. However, the influence of this modeling assumption is limited by the relatively high withdrawal rate from VMB after HIV acquisition as reported in other modeling studies [32]. The strict control on the VMB use by infected individuals will likely prevent the occurrence of drug-resistance which is not included in our model. The difference in HIV risk for pregnant and non-pregnant women in our analysis is based on variation in condom use and per-act susceptibility to HIV between those periods. However, other behavioral factors, such as changes in the frequency and type of sexual activities (anal vs. vaginal sex) may affect the acquisition risks before, during and after pregnancy [33]. The beneficial contribution of providing VMB to pregnant women may be improved or reduced by differential adherence to the product during pregnancy or by variation in the proportion of non-vaginal exposures to HIV which we do not discuss in this paper.

The main conclusion of this study is that providing effective and safe microbicide to pregnant women has public-health significance and must be among the priorities during the development, planning and implementation of wide-scale intervention for HIV prevention.

## Supporting Information

File S1 (DOCX)
