# Supplementary material for: Population-Level Benefits from Providing Effective HIV Prevention Means to Pregnant Women in High Prevalence Settings
Source: PLoS One. 2013 Sep 16;8(9):e73770. doi: 10.1371/journal.pone.0073770 (PMC3774771; doi:10.1371/journal.pone.0073770)
Supplement: File S1 — (DOCX) [file pone.0073770.s001.docx]

**Supporting Information**

**Population-level benefits from providing effective HIV prevention means to pregnant women in high prevalence settings**

Dobromir Dimitrov, Marie-Claude Boily, Jeannie Marrazzo, Richard Beigi, Elizabeth R. Brown

**1. Description and structure of the mathematical model**

Our model is implemented through a system of differential equations which governs the temporal dynamics of the following population compartments:

- $S_{w}^{p} \text{and} S_{w}$ represent pregnant and non-pregnant susceptible women not using VMB, respectively;
- $S_{w}^{Vp} \text{and} S_{w}^{V}$ represent pregnant and non-pregnant susceptible women using VMB, respectively;
- $S_{m}$ - represents susceptible men;
- $I_{w}^{p} \text{and} I_{w}$ represent pregnant and non-pregnant infected women not using VMB, respectively;
- $I_{w}^{Vp} \text{and} I_{w}^{V}$ represent pregnant and non-pregnant infected women using VMB, respectively;
- $I_{m}$ represents infected men;
- $A_{w} \text{and} A_{m}$ represent women and men who developed AIDS, respectively.

The recruitment rates *Λ_w_* and *Λ_m_* into the population are selected to preserve the size of a HIV-free population, i.e., *Λ_w_* =*μN_w_* and *Λ_m_* =*μN_m_* , where $N_{w}=S{}_{w}+S_{w}^{p}+S_{w}^{V}+S_{w}^{Vp}+I{}_{w}+I_{w}^{p}+I_{w}^{V}+I_{w}^{Vp}$ is the number of sexually active women while $N_{m}=S{}_{m}+I{}_{m}$ is the number of sexually active males.

VMB is used by a proportion *k* of the HIV-negative women who reach sexual maturity and enter the sexually active population.

The biological meaning of the parameters and the ranges used in our analysis are given in Table 1.

Model equations which describe the rates of change in the population compartments are:

$$\begin{matrix} \frac{dS_{w}}{dt}=(1-k)\Lambda_{w}+\tau_{2}S_{w}^{p}-\lambda_{w}S_{w}-\tau_{1}S_{w}-\mu S_{w} \\ \frac{dS_{w}^{V}}{dt}=k\Lambda_{w}+\tau_{2}S_{w}^{Vp}-\lambda_{w}^{V}S_{w}^{V}-\tau_{1}S_{w}^{V}-\mu S_{w}^{V} \\ \frac{dS_{w}^{p}}{dt}=\tau_{1}S_{w}-\lambda_{w}^{p}S_{w}^{p}-\tau_{2}S_{w}^{p}-\mu S_{w}^{p} \\ \frac{dS_{w}^{Vp}}{dt}=\tau_{1}S_{w}^{V}-\lambda_{w}^{Vp}S_{w}^{Vp}-\tau_{2}S_{w}^{Vp}-\mu S_{w}^{Vp} \\ \frac{dI_{w}}{dt}=\lambda_{w}S_{w}+\delta I_{w}^{V}+\tau_{2}I_{w}^{p}-\tau_{1}I_{w}-\mu I_{w}-d I_{w} \\ \frac{dI_{w}^{V}}{dt}=\lambda_{w}^{V}S_{w}^{V}-\delta I_{w}^{V}+\tau_{2}I_{w}^{Vp}-\tau_{1}I_{w}^{V}-\mu I_{w}^{V}-d I_{w}^{V} \\ \frac{dI_{w}^{p}}{dt}=\lambda_{w}^{p}S_{w}^{p}+\tau_{1}I_{w}-\tau_{2}I_{w}^{p}-\mu I_{w}^{p}-d I_{w}^{p} \\ \frac{dI_{w}^{Vp}}{dt}=\lambda_{w}^{Vp}S_{w}^{Vp}+\tau_{1}I_{w}^{V}-\tau_{2}I_{w}^{Vp}-\mu I_{w}^{Vp}-d I_{w}^{Vp} \\ \frac{dS_{m}}{dt}=\Lambda_{m}-\lambda_{m}S_{m}-\mu S_{m} \\ \frac{dI_{m}}{dt}=\lambda_{m}S_{m}-\mu I_{m}-d I_{m} \\ \frac{dA_{w}}{dt}=d(I_{w}+I_{w}^{p}+I_{w}^{V}+I_{w}^{Vp}) \\ \frac{dA_{m}}{dt}=dI_{m} \end{matrix}$$

where $d$is the departure rate from sexually active population due to HIV including HIV related mortality and progression to AIDS, $\delta$is the rate at which HIV-positive users of VMB withdraw from the product, $1/\mu$ is the average time to remain sexually active, $\tau_{1}$ is the annual pregnancy rate (proportion of women who become pregnant during one year), $\tau_{2}$ is the annual rate of return from pregnancy.

The forces of infections ($\lambda_{w},\lambda_{m},\lambda_{w}^{p},\lambda_{w}^{V}, and \lambda_{w}^{Vp}$) are based on the number of sex partners per year (*ρ*), annual HIV acquisition risks in serodiscordant partnerships when the female partner is pregnant or not and is using VMB or not and the fraction of the opposite gender which is currently infected:

$$\begin{matrix} \lambda_{w}=\rho R_{w}\frac{I{}_{m}}{N_{m}} \\ \lambda_{w}^{V}=\rho R_{w}^{V}\frac{I{}_{m}}{N_{m}} \\ \lambda_{w}^{p}=\rho R_{w}^{p}\frac{I{}_{m}}{N_{m}} \\ \lambda_{w}^{Vp}=\rho R_{w}^{Vp}\frac{I{}_{m}}{N_{m}} \\ \lambda_{m}=\rho(R_{m}\frac{I{}_{w}}{N_{w}}+R_{m}^{V}\frac{I_{w}^{V}}{N_{w}}+R_{m}^{p}\frac{I_{w}^{p}}{N_{w}}+R_{m}^{Vp}\frac{I_{w}^{Vp}}{N_{w}}) \\ R_{w}=1-(1-(1-\alpha_{c})\beta_{w})^{cn_{w}/\rho}(1-\beta_{w})^{(1-c)n_{w}/\rho} \\ R_{w}^{V}=1-(1-(1-\alpha_{c})(1-\alpha_{s})\beta_{w})^{cn_{w}/\rho}(1-(1-\alpha_{s})\beta_{w})^{(1-c)n_{w}/\rho} \\ R_{w}^{p}=1-(1-(1-\alpha_{c})r_{p}\beta_{w})^{\gamma cn_{w}/\rho}(1-r_{p}\beta_{w})^{(1-\gamma c)n_{w}/\rho} \\ R_{w}^{Vp}=1-(1-(1-\alpha_{c})(1-\alpha_{s})r_{p}\beta_{w})^{\gamma cn_{w}/\rho}(1-(1-\alpha_{s})r_{p}\beta_{w})^{(1-\gamma c)n_{w}/\rho} \\ R_{m}=1-(1-(1-\alpha_{c})\beta_{m})^{cn_{m}/\rho}(1-\beta_{m})^{(1-c)n_{m}/\rho} \\ R_{m}^{V}=1-(1-(1-\alpha_{c})(1-\alpha_{i})\beta_{m})^{cn_{m}/\rho}(1-(1-\alpha_{i})\beta_{m})^{(1-c)n_{m}/\rho} \\ R_{m}^{p}=1-(1-(1-\alpha_{c})r_{p}\beta_{m})^{\gamma cn_{m}/\rho}(1-r_{p}\beta_{m})^{(1-\gamma c)n_{m}/\rho} \\ R_{m}^{Vp}=1-(1-(1-\alpha_{c})(1-\alpha_{i})r_{p}\beta_{m})^{\gamma cn_{m}/\rho}(1-(1-\alpha_{i})r_{p}\beta_{m})^{(1-\gamma c)n_{m}/\rho} \end{matrix}$$

where $\beta_{w}$($\beta_{m}$) is the HIV acquisition risk for non-pregnant women (men) per unprotected vaginal sex act, $r_{p}$ is the relative HIV acquisition risk per act for men and women during pregnancy, $n_{w}(n_{m})$ is the average number of sex acts per year for women (men), $\alpha_{s} (\alpha_{i})$ is the efficacy of VMB in reducing susceptibility (infectiousness) of the user per vaginal sex act, $\alpha_{c}$ is the condom efficacy per sex act, $c$ is the rate of condom use in general population and $\gamma$ is the relative rate of condom use when the female partner is pregnant. The risks for women and men in serodiscordant partnerships to acquire HIV are denoted as follows:

$R_{w}$and $R_{m}$ when the female partner is not pregnant and she is not using VMB,

$R_{w}^{p}$and $R_{m}^{p}$ when the female partner is pregnant and she is not using VMB,

$R_{w}^{V}$and $R_{m}^{V}$ when the female partner is not pregnant and she is using VMB,

$R_{w}^{Vp}$and $R_{m}^{Vp}$ when the female partner is pregnant and she is using VMB

VMB interventions are initiated in populations of 1,000,000 women and same number of men (*N_m_*(0)=*N_w_*(0)=1000000) under epidemic conditions representative for South Africa. The initial size of the population compartments is set as follows:

$$\begin{matrix} S_{w}(0)=(1-k_{1})(1-p)(1-P_{w})N_{w}(0) \\ S_{w}^{V}(0)=k_{1}(1-p)(1-P_{w})N_{w}(0) \\ S_{w}^{p}(0)=(1-k_{1})p(1-P_{w})N_{w}(0) \\ S_{w}^{Vp}(0)=k_{1}p(1-P_{w})N_{w}(0) \\ S_{m}(0)=(1-P_{m})N_{m}(0) \\ I_{w}(0)=(1-(1-\theta)k_{1})(1-p)P_{w}N_{w}(0) \\ I_{w}^{V}(0)=(1-\theta)k_{1}(1-p)P_{w}N_{w}(0) \\ I_{w}^{p}(0)=pP_{w}N_{w}(0) \\ I_{w}^{Vp}(0)=0 \\ I_{m}^{p}(0)=P_{m}N_{m}(0) \end{matrix}$$

VMB is initially used by a proportion *k_1_* of the HIV-negative and by a reduced proportion of (1-θ) *k_1_* of the HIV-positive women due to pre-enrollment HIV screening. Here $\theta$represents the prescription rejection rate which measures the reduction in the initial fraction of HIV-positive compared to HIV-negative women who start using VMB (pre-enrollment control) while $p$ is the initial fraction of women who are pregnant

**2. Cumulative HIV acquisition risk over fixed periods in absence of VMB**

The cumulative female risk over a non-pregnant period (assuming 100% of sex acts with a HIV positive partner) is:

where $\beta_{w}$ is the HIV acquisition risk for non-pregnant women per unprotected vaginal sex act,  *c* is the fraction of sex acts protected by condoms, $\alpha_{c}$ is the condom efficacy per sex act, and *n* is the number of sex acts for the period.

The cumulative female risk over a pregnant period (assuming 100% of sex acts with a HIV positive partner) is:

where $r_{p}$ is the relative HIV acquisition risk per act during pregnancy and $\gamma$ is the relative rate of condom use when the female partner is pregnant compared to non-pregnant.

The relative HIV acquisition risk (RR_HIV_) during pregnancy compared to non-pregnant period, presented in the main text (Fig. 2A), is calculated as:

using parameter values of $\beta_{w}=0.3\%$ *, c=50%,* $\alpha_{c}=90\%$and *n=60*. The same level of sexual activity is assumed before and during pregnancy.

**3. Additional results**


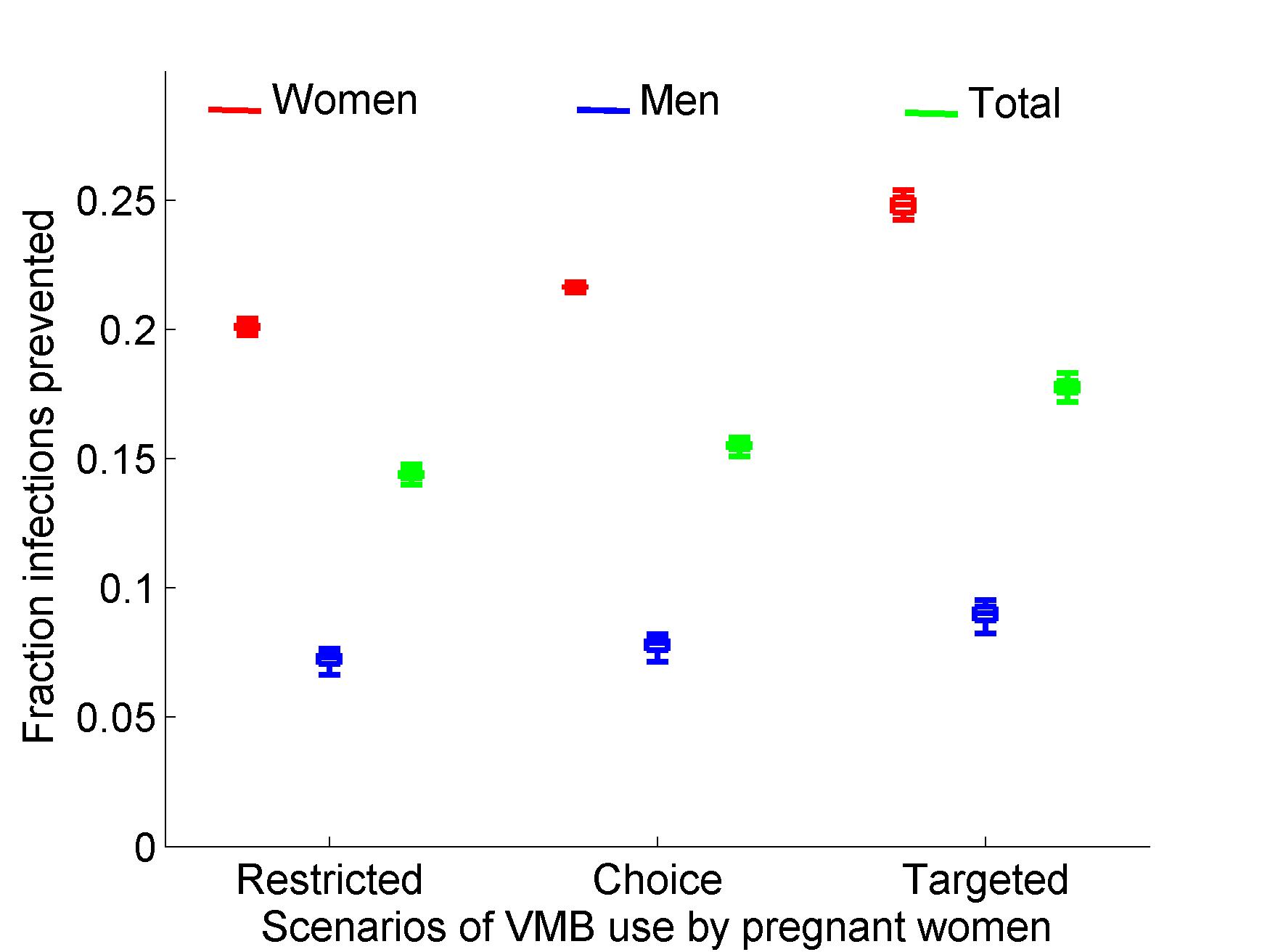


A)


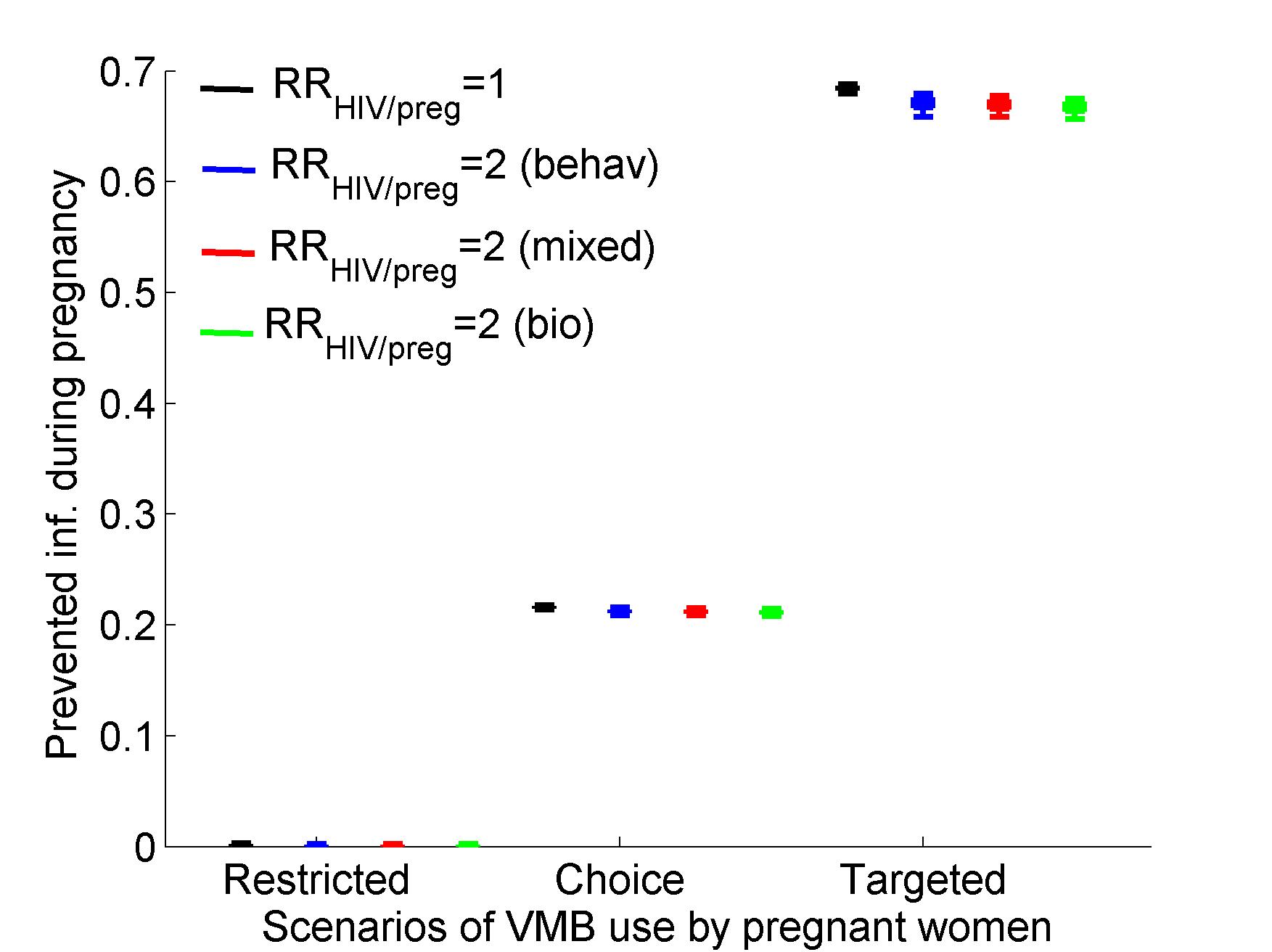

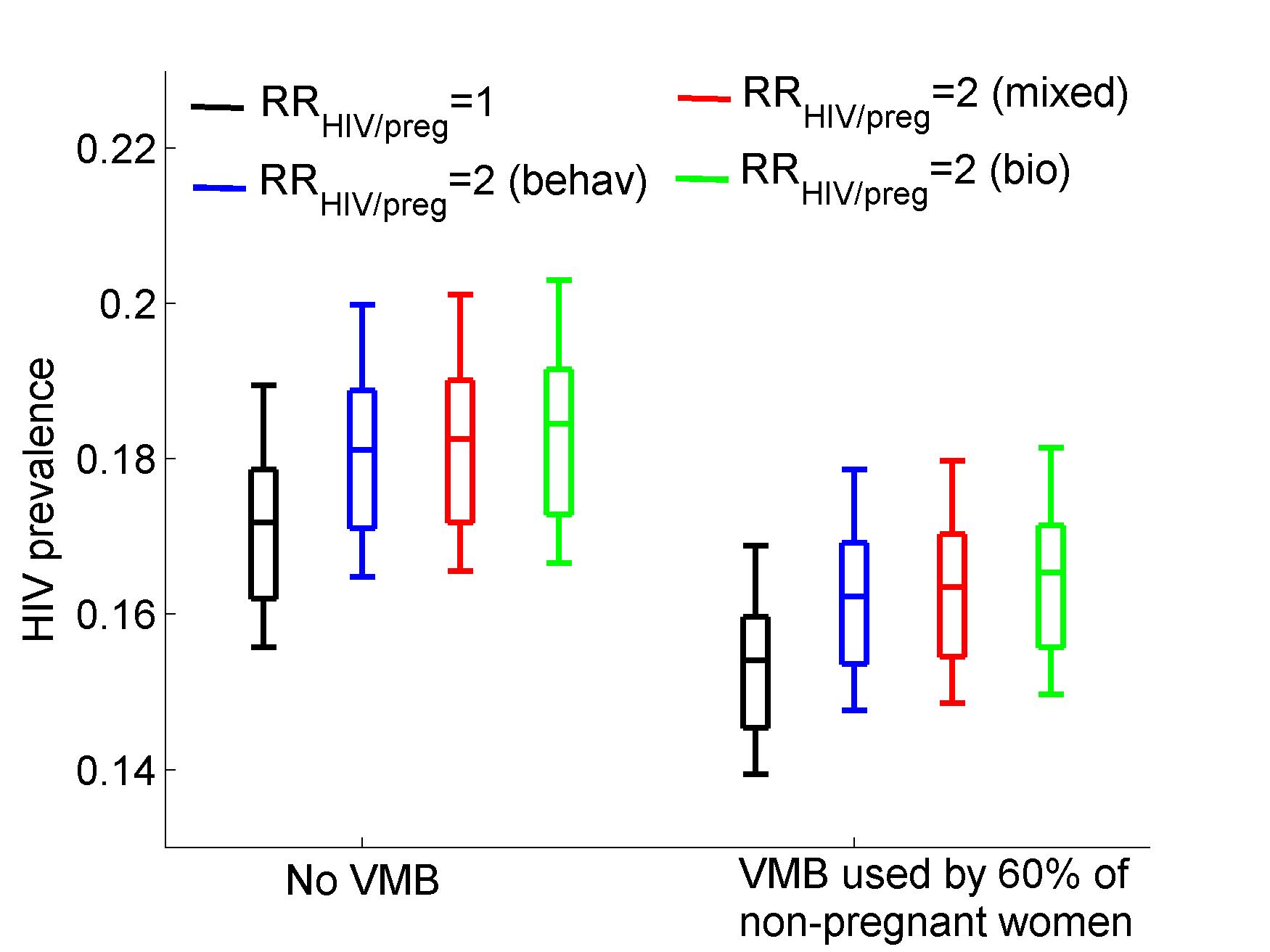

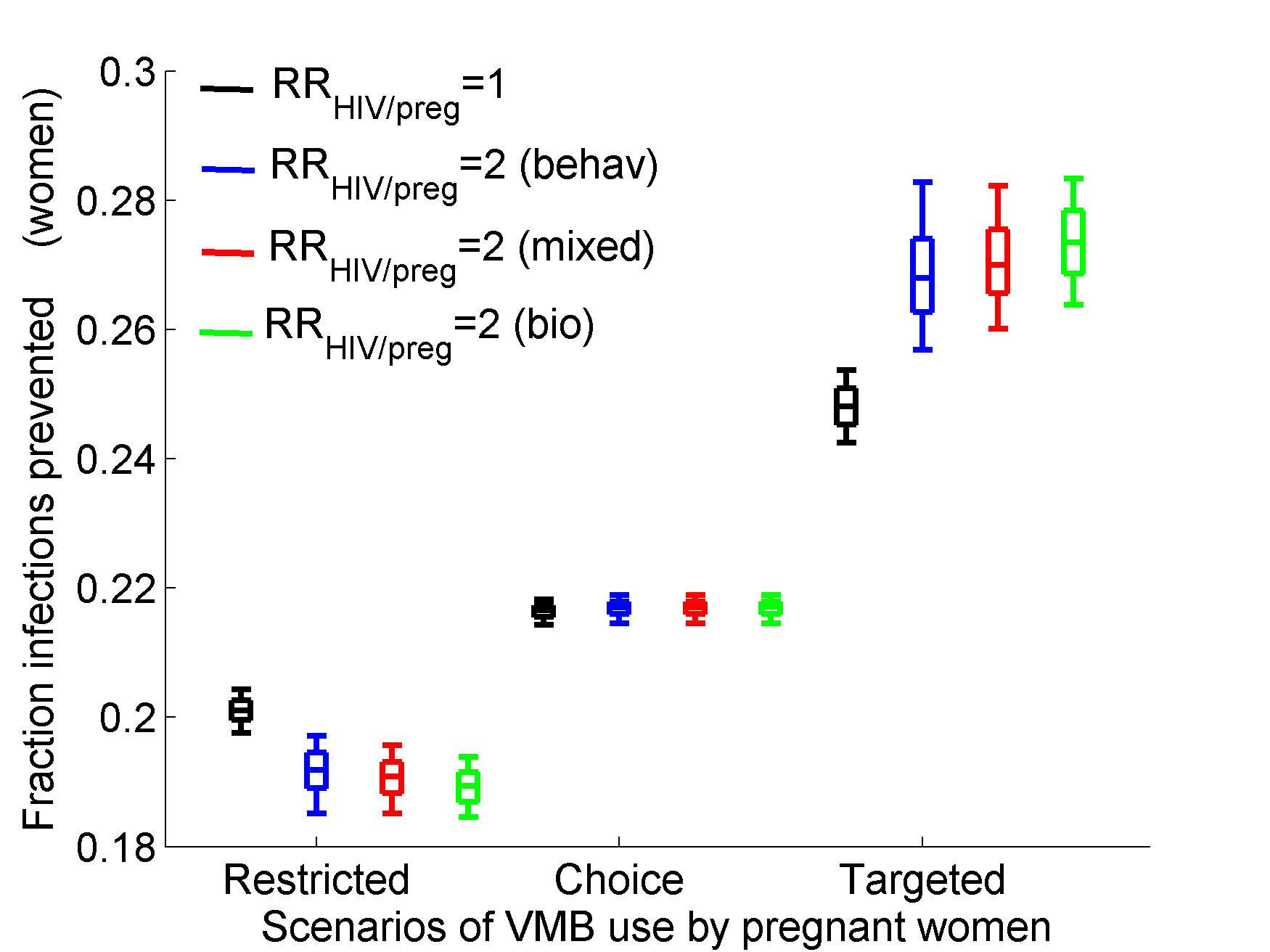


B)

C)

D)

**Figure S1.** Comparison of the impact of interventions with 70% efficacious VMB used by 30% of the non-pregnant women under different scenarios of VMB use by pregnant women: A) Cumulative fraction of infections prevented over 10 years in women (red), men (blue) and total (green) assuming no change in HIV risk during pregnancy (RR_HIV/preg_=1) ; B) Cumulative fraction of infections prevented in women; C) Projected HIV prevalence after 10 years assuming no VMB use and VMB use by non-pregnant women only. D) Cumulative fraction of infections during pregnancy prevented over 10 years. The scenarios with elevated risk during pregnancy use parameter combinations described in Fig.2A. The box plots (median, 5th, 25th, 75th, 95th percentiles) reflect the variation in estimates generated by 1,000 different epidemic sets.


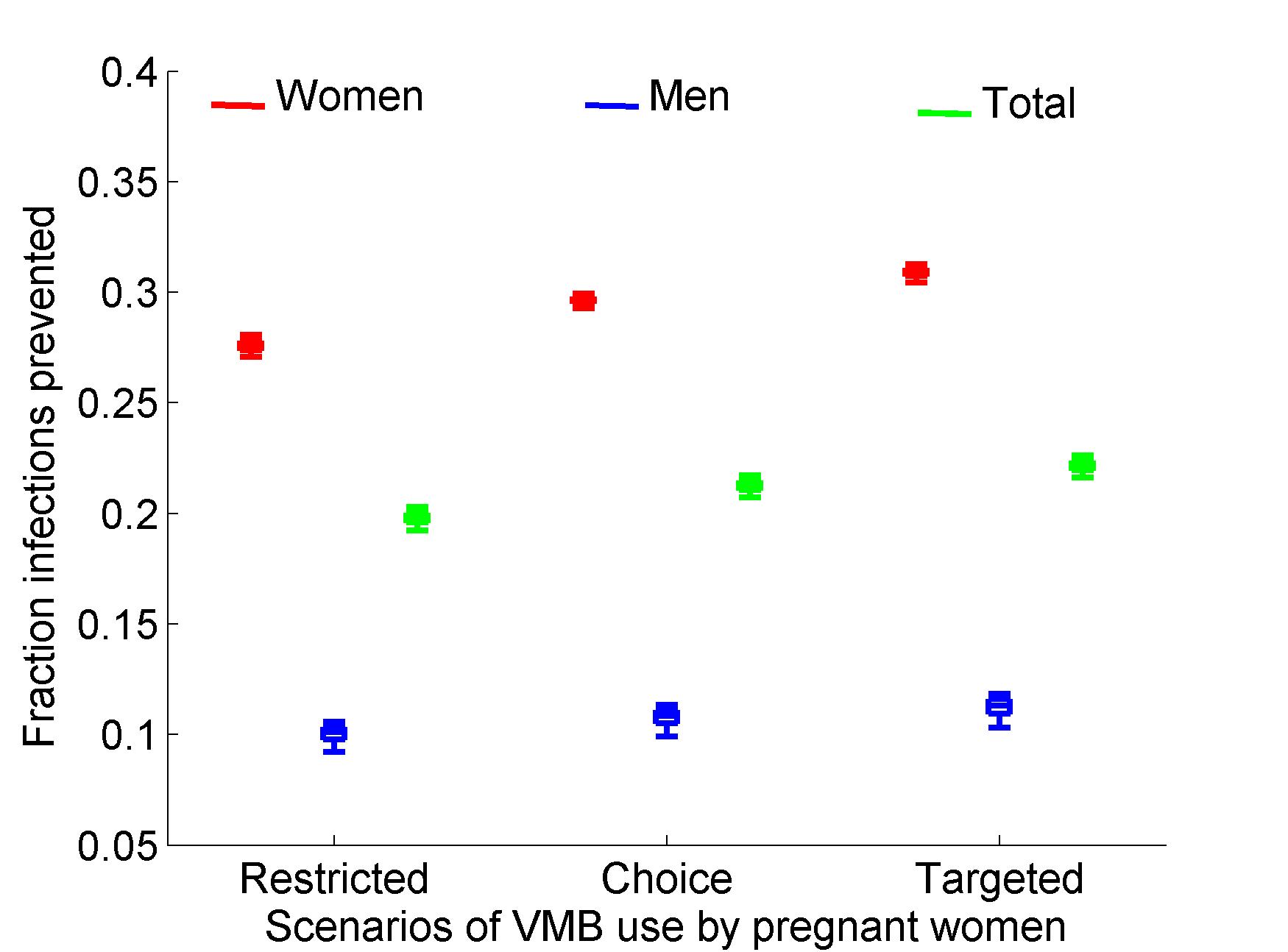


A)


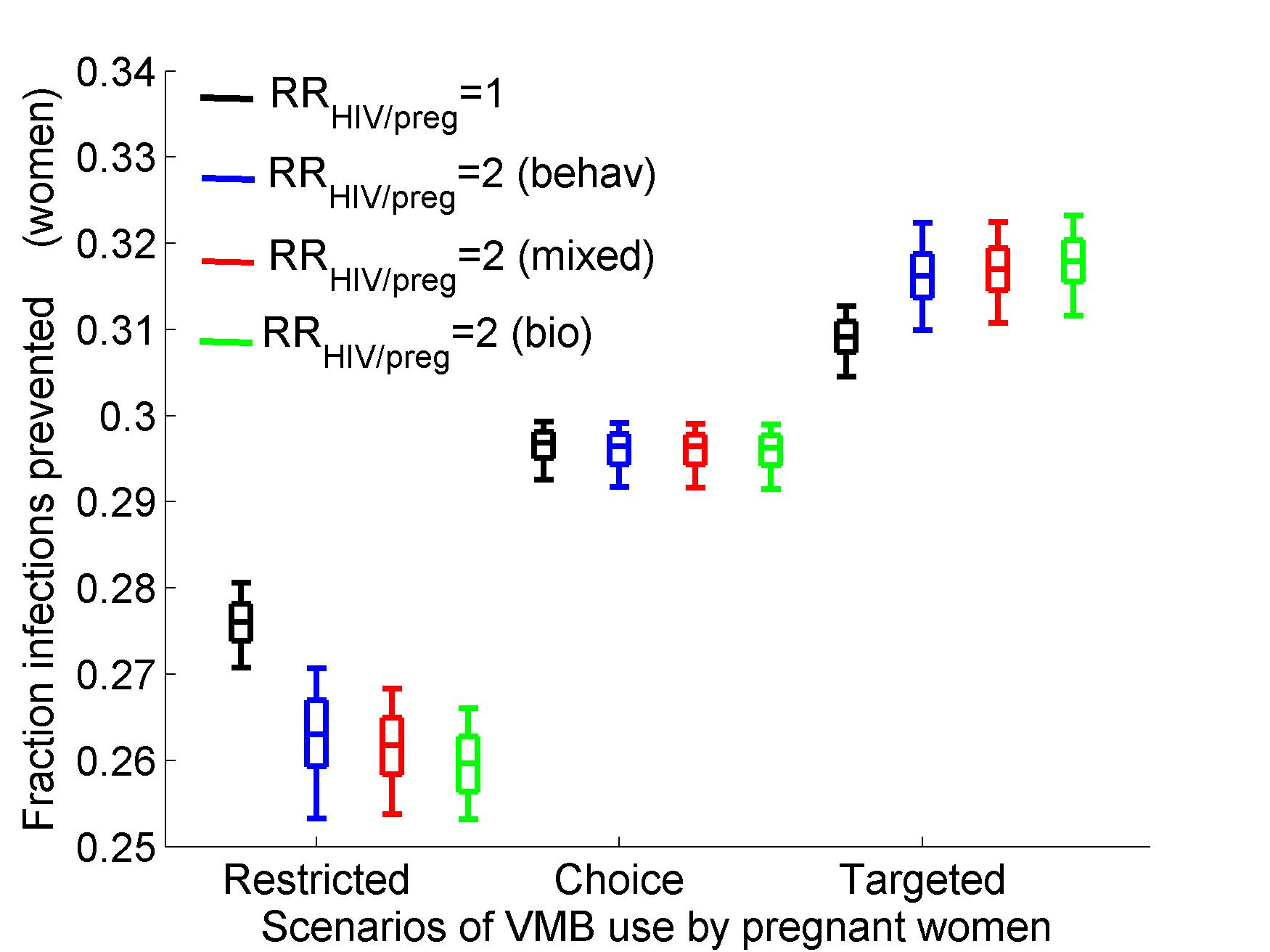

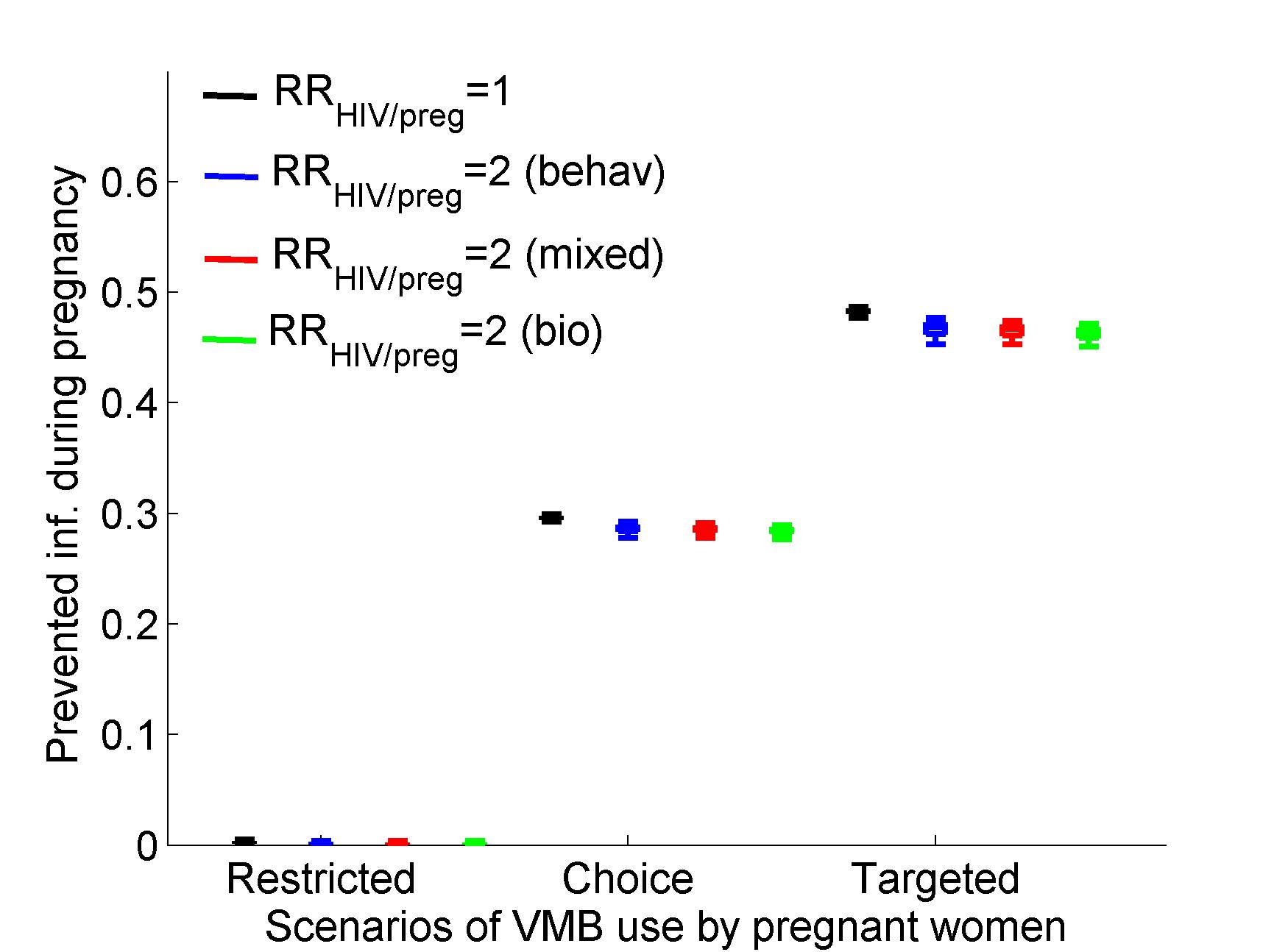

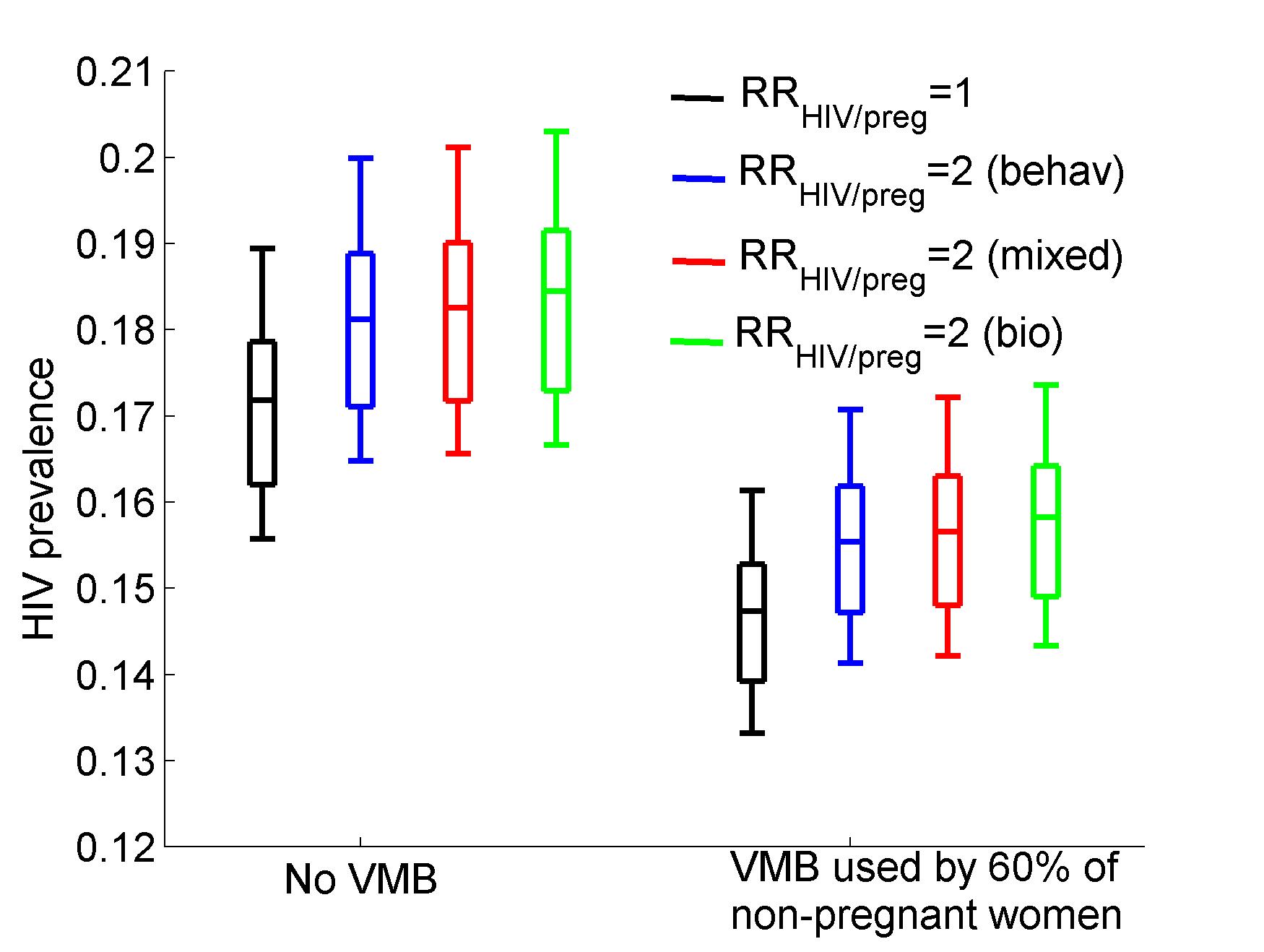


B)

C)

D)

**Figure S2.** Comparison of the impact of interventions with 50% efficacious VMB used by 60% of the non-pregnant women under different scenarios of VMB use by pregnant women : A) Cumulative fraction of infections prevented over 10 years in women (red), men (blue) and total (green) assuming no change in HIV risk during pregnancy (RR_HIV/preg_=1) ; B) Cumulative fraction of infections prevented in women; C) Projected HIV prevalence after 10 years assuming no VMB use and VMB use by non-pregnant women only. D) Cumulative fraction of infections during pregnancy prevented over 10 years. The scenarios with elevated risk during pregnancy use parameter combinations described in Fig.2A. The box plots (median, 5th, 25th, 75th, 95th percentiles) reflect the variation in estimates generated by 1,000 different epidemic sets


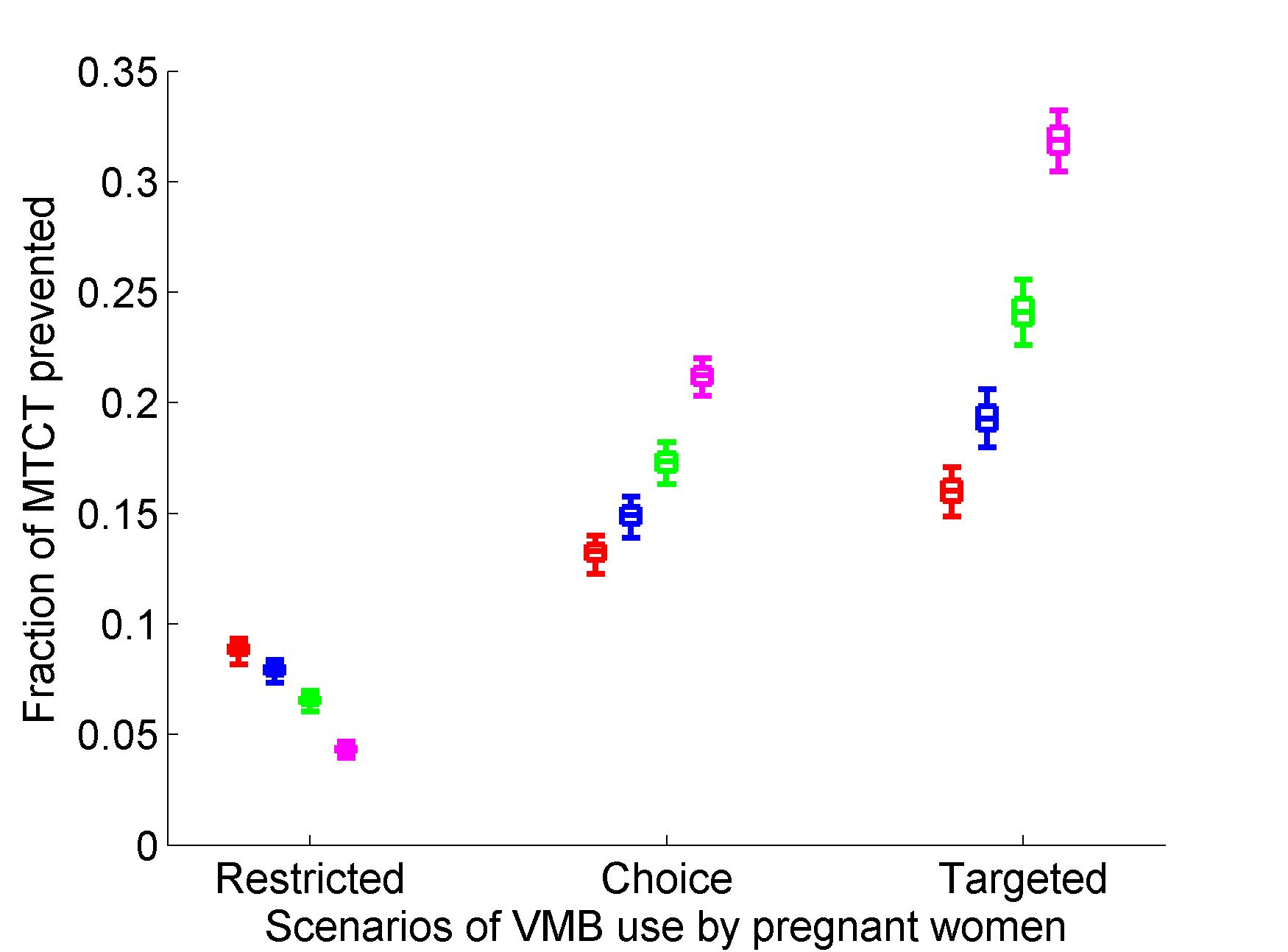

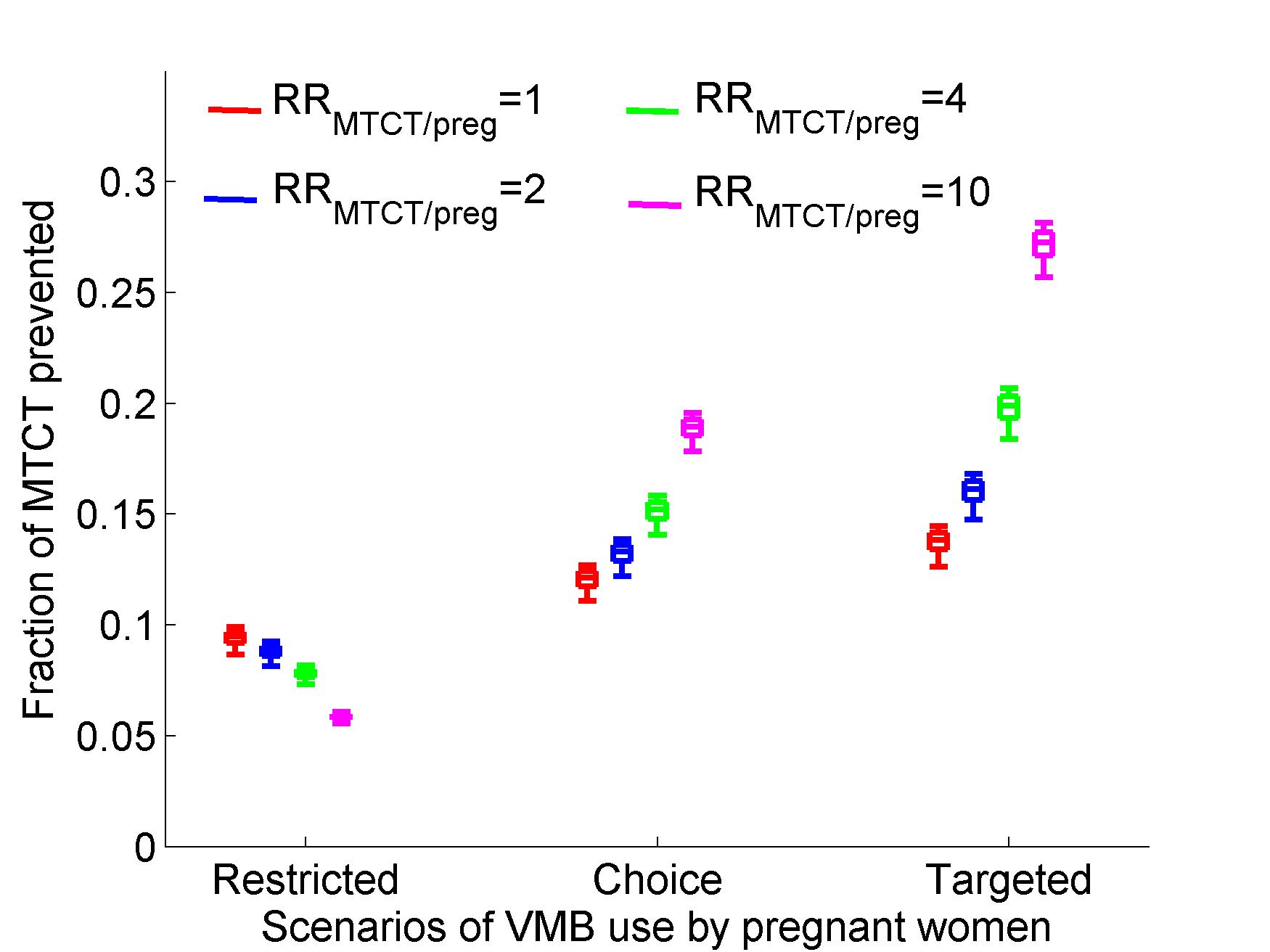


A)

B)

**Figure S3.** Cumulative fraction of MTCT prevented (right) over 10 years by intervention with 50% efficacious VMB used by 60% of the non-pregnant women under different scenarios of VMB usage by pregnant women assuming: A) no change in HIV risk during pregnancy (RR_HIV/preg_=1) and B) HIV risk during pregnancy is elevated (RR_HIV/preg_=2, mixed scenario). The colors represent different level of the relative risk of MTCT (RR_MTCT/preg_ ) when HIV is acquired during pregnancy compared to when acquired before pregnancy. The box plots (median, 5th, 25th, 75th, 95th percentiles) reflect the variation in estimates generated by 1,000 different parameters sets.

**4. Sensitivity analysis**

The influence of other intervention assumptions on the intervention outcomes is studied in a multivariate sensitivity analysis and the correlations between input and output parameters are presented in Figure S4. The VMB protection against HIV (reduced susceptibility) and VMB coverage show the strongest positive correlation with the fraction of infections prevented in men and women and the reduction in the residue MTCT due to VMB use over 10 years (Fig. S4). When all pregnant women use VMB, infections prevented during pregnancy depend on the reduced susceptibility by VMB but not on the VMB coverage among non-pregnant women (Fig. S4 A). Conversely, if none of the pregnant women use VMB, the VMB coverage has stronger influence than reduced susceptibility on the HIV acquisition during pregnancy (Fig. S4 C).Theoretical reduction in infectiousness of HIV positive VMB users may be more important for that outcome since it affects the number of male infections and indirectly reduces the acquisition risk for pregnant women. Note that the influence of the VMB coverage on the prevented MTCT decreases with the increase of the relative risk of vertical transmission when HIV is acquired during the prenatal period (Fig. S4 B). The efforts to restrict VMB use by infected women through initial and periodic HIV testing may have small negative impact on the intervention outcomes but may have significant role in preventing the spread on drug-resistance.


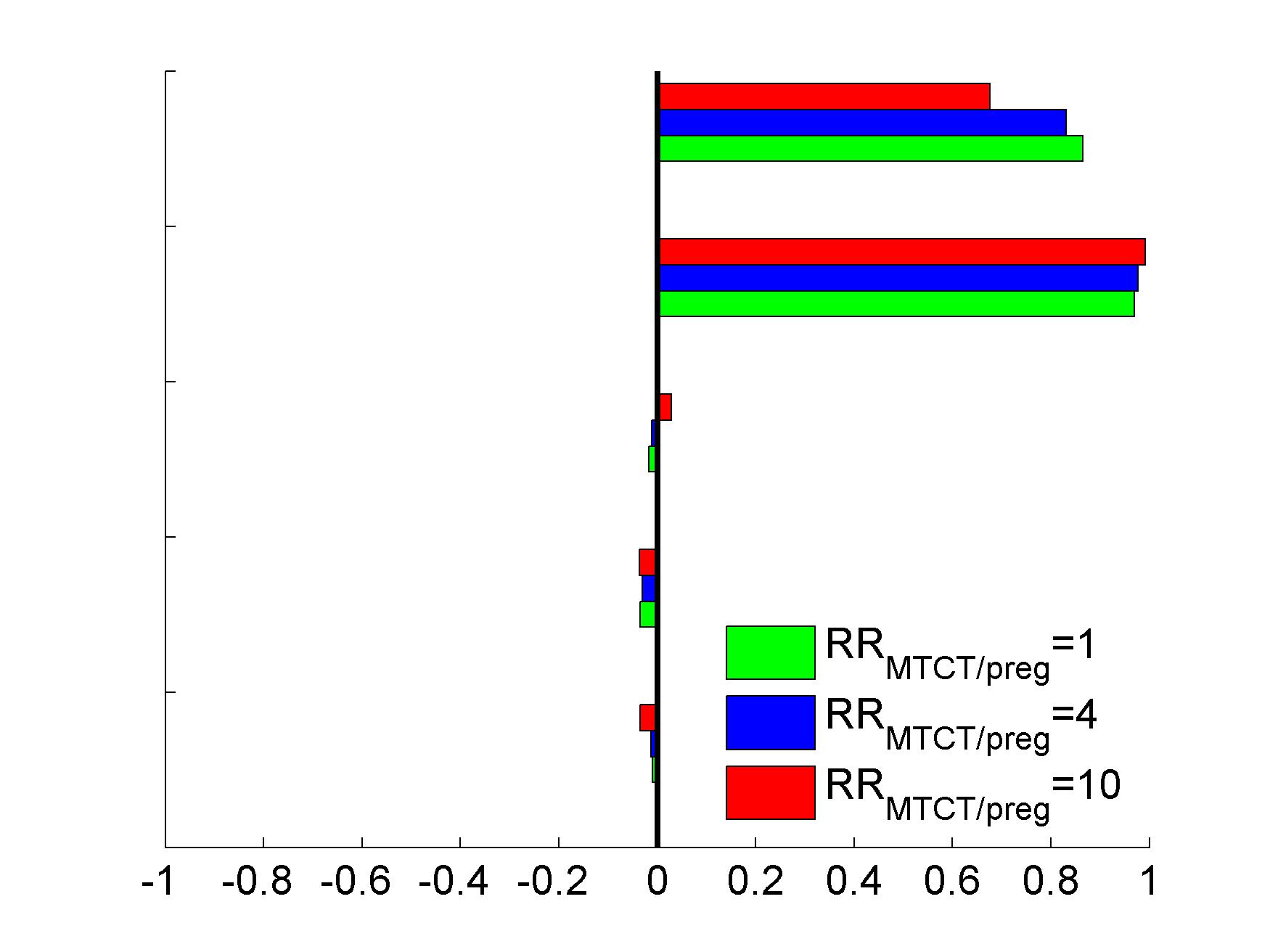

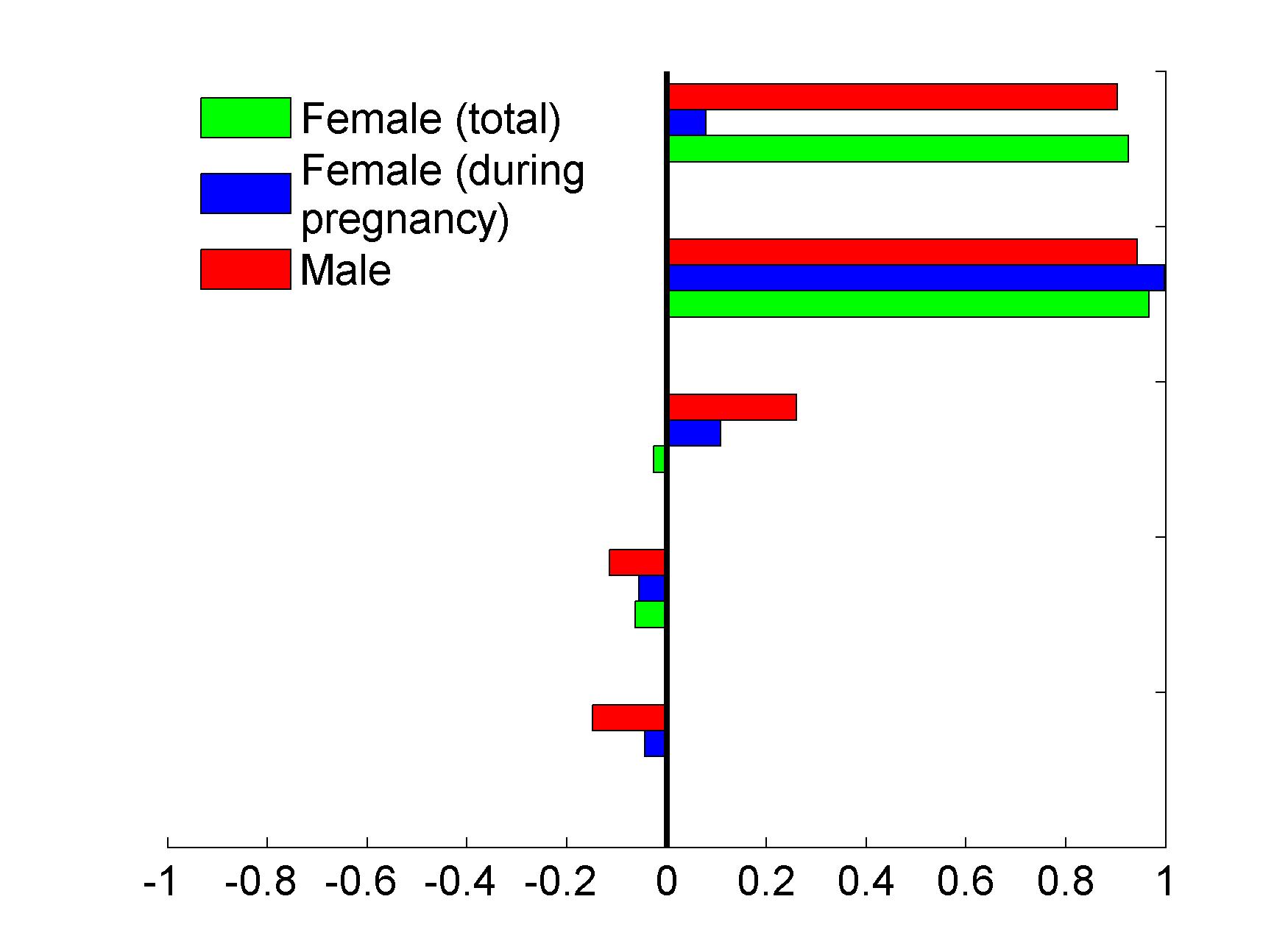

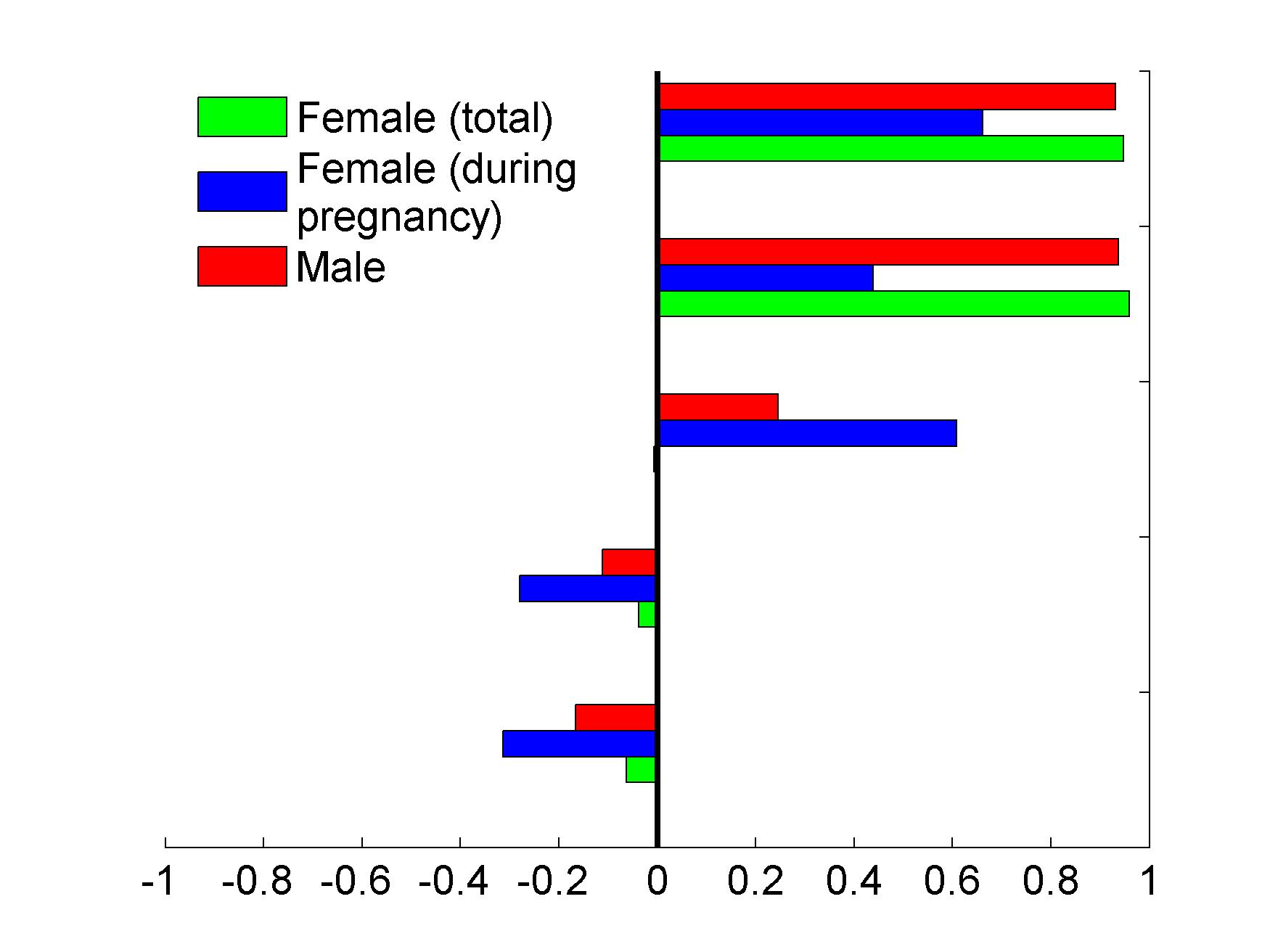

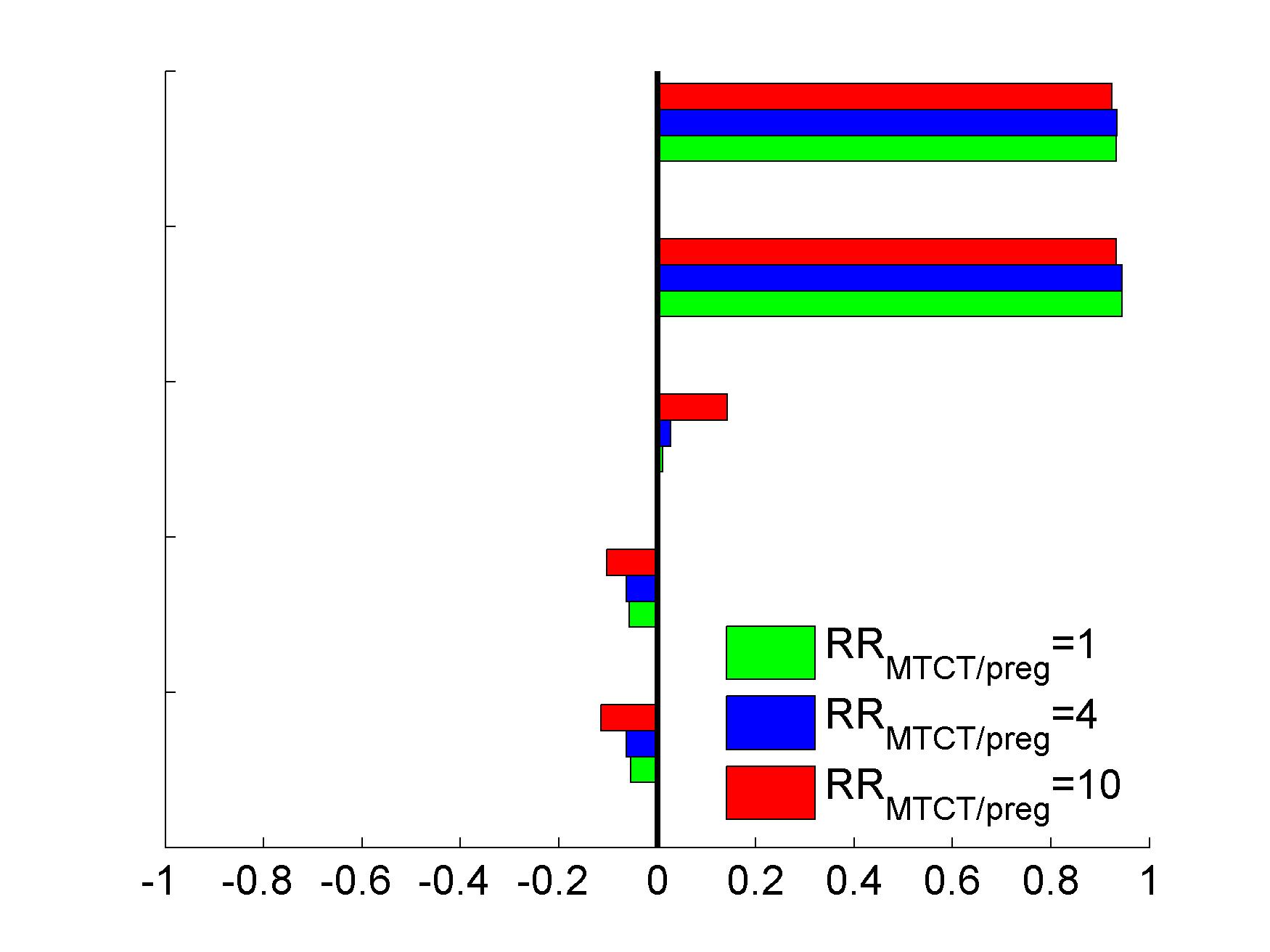


Intervention parameters:

VMB coverage (percentage of

non-pregnant women using VMB)

VMB efficacy in reducing susceptibility

VMB efficacy in reducing infectiousness

Prescription rejection rate for HIV

positive women who want to start

using VMB

 VMB withdrawal rate when HIV positive

Intervention parameters:

VMB coverage (percentage of

non-pregnant women using VMB)

VMB efficacy in reducing susceptibility

VMB efficacy in reducing infectiousness

Prescription rejection rate for HIV

positive women who want to start

using VMB

 VMB withdrawal rate when HIV positive

Correlation with infections prevented

Correlation with MTCT prevented

A)

B)

C)

D)

Correlation with infections prevented

Correlation with MTCT prevented

All pregnant women use VMB

None of the pregnant women use VMB

**Figure S4.** Partial rank correlation coefficients (PRCC) between intervention parameters and outcomes assuming that A), B) all uninfected pregnant women.use VMB and C), D) none of the uninfected pregnant women.use VMB. Correlations presented are between input parameters and A), C) the cumulative fraction of infections prevented in men (red), in women total (green) and in women during pregnancy (blue) over 10 years of VMB use; B), D) the fraction of the residue MTCT prevented over 10 years of VMB use under different assumption regarding the relative risk from MTCT when the HIV is acquired during pregnancy. Based on 10, 000 simulations (10 per preselected epidemic set).
